# Exploring the Feasibility and Effects of a Ketogenic Diet in Patients With CNS Malignancies: A Retrospective Case Series

**DOI:** 10.3389/fnins.2020.00390

**Published:** 2020-05-19

**Authors:** Cristina M. Panhans, Gillian Gresham, L. J. Amaral, Jethro Hu

**Affiliations:** Cedars-Sinai Medical Center, Samuel Oschin Comprehensive Cancer Center, Los Angeles, CA, United States

**Keywords:** ketogenic diet, glioma, glioblastoma, astrocytoma, Warburg effect, Atkins, ketosis, metabolic therapy

## Abstract

**Background:** Recently, the ketogenic diet has been proposed as an adjunct treatment for a range of medical conditions including weight loss, diabetes, cancer, and neurodegenerative diseases. Because malignant CNS tumors are highly dependent on glucose, the use of a ketogenic diet as an adjunct therapy is currently being explored. This case series summarizes our experience implementing a ketogenic diet for patients with CNS malignancies.

**Methods:** Patients diagnosed with CNS malignancies following a ketogenic diet were identified between 2015 and 2017. Malignancies included confirmed diagnoses of glioblastoma (GBM), astrocytoma, or oligodendroglioma. With guidance from a registered dietitian, ketone levels, glucose levels, and weight were regularly collected for several patients along with patient-reported symptoms and adverse effects. Interested patients were asked to follow a 3:1 ketogenic diet for 120 days. The ketogenic diet is a high-fat, moderate protein, and very low carbohydrate diet, where patients limited carbohydrate intake to ≤20 g per day. Brain imaging was reviewed. A series of descriptive analyses were conducted.

**Results:** The ketogenic diet was initiated in 12 patients of which 8 patients contributed data on their blood glucose and ketone levels. The majority of patients were male (*n* = 10) with a median age of 45 (range 32–62). Diagnoses included GBM (*n* = 6), grade 2/3 astrocytomas (*n* = 5) and one patient with a grade 2 spinal cord astrocytoma. Ten of the 12 patients were receiving concurrent treatment; two received supportive care only. The majority of patients with evaluable data (*n* = 8) maintained ketone levels above 0.5 mM for the duration of 120-day period. Ketone levels generally increased from baseline while glucose levels and BMI decreased. Overall, patients reported improved symptoms over the course of the diet. Imaging also suggested improved disease control and reduction in vasogenic edema.

**Conclusion:** Taking advantage of a tumor’s metabolic inflexibility can have a positive impact on patients, particularly those with CNS malignancies. More structured and statistically planned clinical trials are needed to determine the margin of impact of a ketogenic diet.

## Introduction

CNS malignancies are devastating diagnoses, and current therapies leave much to be desired. Standard treatment often consists of surgery, radiation therapy, and chemotherapy; these therapies are generally not curative, and in many cases prolongation of survival is modest at best. For GBM – the most common primary malignant brain tumor – median overall survival is approximately 15–18 months (Ostrom et al., 2014; Woolf and Scheck, 2015; Nabors et al., 2017). Lower grade gliomas (i.e., WHO grade 2 and grade 3 glioma, as opposed to grade 4 glioma which is synonymous with GBM) carry better prognoses, but not by much. In adults, depending on grade and tumor subtype (e.g., oligodendroglioma vs. astrocytoma, *IDH1-*mutant vs. *IDH1-*wild-type) survival can range from a months to over a decade (Scorsetti et al., 2015; Society, 2017). Unfortunately, the vast majority of adults diagnosed with a glioma of any grade ultimately succumb to the disease.

Given this context, it is understandable that many patients with CNS malignancies often seek complementary or alternative therapies, including dietary intervention. One such intervention gaining attention in the cancer community is the ketogenic diet (Zhou et al., 2007; Paoli et al., 2011; Schmidt et al., 2011; Khodadadi et al., 2017; Klement, 2017). The ketogenic diet is an established and effective treatment for epilepsy, with use for refractory pediatric epilepsy dating back to the 1920s (Hartman and Vining, 2007). More recently, the ketogenic diet has been proposed as an adjunct treatment for a range of medical conditions including diabetes, weight loss, cancer, psychiatric, and neurodegenerative diseases (Hartman and Vining, 2007; Rho and Stafstrom, 2012; Hull et al., 2016).

The ketogenic diet is a high fat, moderate protein, and low carbohydrate diet that results in the endogenous production of ketones. Unlike other tissues, the CNS is normally solely dependent on glucose as a source of energy; when glucose availability is limited, ketones are metabolized as an alternative energy source. Additionally, ketones serve as substrates for biosynthetic reactions and have cell signaling function (Zhou et al., 2007). The effects of a ketogenic diet are manifold, and theories abound as to its potential anti-neoplastic properties (Khodadadi et al., 2017). Chief among them is the idea that the reliance – and perhaps “addiction” – of cancer cells on glucose and glycolysis renders them susceptible to strategies that reduce glucose availability (Gatenby and Gillies, 2004; Kroemer and Pouyssegur, 2008). This observation of a cancer cell’s usage of aerobic glycolysis is known as the Warburg effect (Xie et al., 2014; Meidenbauer et al., 2015; Woolf and Scheck, 2015; Strickland and Stoll, 2017). Up to 60–80% of cancers exhibit the Warburg effect; however, brain tumors tend to exhibit the Warburg effect at higher rates (Xie et al., 2014). As cancer cells are dependent on abundant levels of glucose, a ketogenic diet may put the cancer cells under metabolic stress and inhibit or delay the cancer’s growth (Champ et al., 2014; Seyfried et al., 2015). Other studies point to potential anti-angiogenic and anti-proliferative effects, decreased substrates for macromolecular synthesis, as well as possible effects on oxidative stress, the NLRP3 inflammasome, and the regulation of gene expression (Johnson et al., 2007; Jarrett et al., 2008; Cotter et al., 2013; Woolf and Scheck, 2015; Youm et al., 2015; Hull et al., 2016). However, clinical data from actual patients with CNS malignancies is limited.

In our institution, the groundswell of interest in the ketogenic diet helped spur a collaboration between patients, oncology dietitians, and neuro-oncologists. In March 2015 we launched a pilot project for patients that included close dietary supervision with regular monitoring of glucose and ketone levels, body weight and composition, and clinical outcomes. Patients who participated in the initial project were also given the opportunity to use proprietary prepared ketogenic meals as part of their meal plans. As interest in the ketogenic diet continues to grow, our clinical practice has now shifted to discussing the potential risks and benefits of the diet with interested patients on a regular basis, with careful emphasis on the lack of robust clinical evidence to date. The data we have collected is presented here as a retrospective analysis.

## Materials and Methods

### Patients

We identified 12 patients diagnosed with CNS malignancies seen at Cedars-Sinai Medical Center between 2015 and 2018 who saw a registered dietitian. Malignancies included a confirmed diagnosis of GBM, astrocytoma, and oligodendroglioma. This study summarizes treatment charts in a retrospective analysis. We extracted data including demographics, clinical diagnosis, MRI scans, blood, glucose and ketone levels, weight, clinical presentations, and adverse events from patient charts between March 1, 2015 through March 5, 2018 (data cut-off date).

All activities were conducted with prior approval and oversight from Cedars-Sinai Medical Center’s Institutional Research Board and Office of Research Compliance and Quality Improvement.

### Description of Ketogenic Diet

A registered dietitian advised patients on ketogenic diet implementation. Meals were provided by Epigenix Foundation to eight patients (cohort 1) for the first 30 days. Meals were provided on a weekly basis with the opportunity to modify meal plans at weekly intervals. Patients also had flexibility with snacks with guidance from the dietitian. Regardless of modification, meal plans aimed for about 20% of total calories from protein, about 7% of calories from carbohydrates, and 73% (minimum) of calories from fat. The remaining patients received meal plans only (cohort 2). Interested patients were instructed to follow a 3:1 ketogenic diet for 120 days. Patients limited carbohydrate intake (including breads, rice, fruits, vegetables, and some dairy products) to ≤20 g of net carbohydrates per day.

A 3:1 ketogenic diet is accepted as a “classic” ketogenic diet for adults. This ratio refers to the amount of fat grams included relative to the net carbohydrate plus protein grams. Thus, a classic ketogenic diet aims for approximately 82% of total calories coming from fat. The purpose of a ketogenic diet is to generate a continuous state of ketosis, meaning low blood glucose and high circulating blood ketones. Although levels of ketones and glucose fluctuate throughout the day, ideally ketones would be present throughout the duration of the diet.

Blood ketone and glucose levels were measured using the Precision Xtra Meter. Patients were trained how to use the Precision Xtra Meter and asked to measure these values every day in the morning and evening prior to eating. Several patients already self-initiated some variation of the diet prior to deciding to using the Precision Xtra for daily measurements. Thus, the baseline for most patients only reflected the day prior to our instructed guidelines for the ketogenic diet. Total daily ketone (D-βHB only) and glucose levels as measured by the Precision Xtra Meter were self-reported by eight patients into a database that generated daily reports to the neuro-oncologist and dietitian. Patients took pictures of their meters for confirmation. Four patients took measurements using urine strips; this data was excluded from quantitative analysis. Ketosis was defined as maintaining average blood ketone levels >0.5 mM for the duration of the data collection period (120 days). This threshold is determined based on previous studies that have evaluated the ketogenic diet in advanced cancers (Schmidt et al., 2011). Blood ketone and glucose levels were measured in mM and mg/dL, respectively, and summarized as daily averages if more than one measurement was obtained. Glucose and ketone levels were still included in analysis if taken at different times than advised. The GKI was calculated by dividing glucose by ketones to obtain a ratio of glucose to ketone levels in the blood (Meidenbauer et al., 2015).

Body mass index was calculated based on weight and kg following the kg/m^2^ formula provided in the patient charts or dietitian progress notes. All symptom and survival data were retrospectively extracted from clinician and dietitian notes in the medical chart. When reported, symptoms were summarized at baseline, 30 days, 60 days, and end of study including: fatigue, cognitive function, nausea/vomiting, constipation, energy, mood, and seizure control. A qualitative summary of patient symptom improvement was obtained based on reports from the treating providers. When available, the ESAS (Bruera et al., 1991), routinely collected as per our institutional standards, was used to measure symptoms at beginning and end of study. Disease progression was assessed using MRIs and radiologist report.

### Analysis

As this was a retrospective case series, data were not aggregated or summarized across patients. Descriptive and graphical summaries of individual-level patient data were provided in evaluable patients including BMI, glucose and ketone levels, and GKI. All analyses were completed using Stata (v15.0).

## Results

**FIGURE 1:**
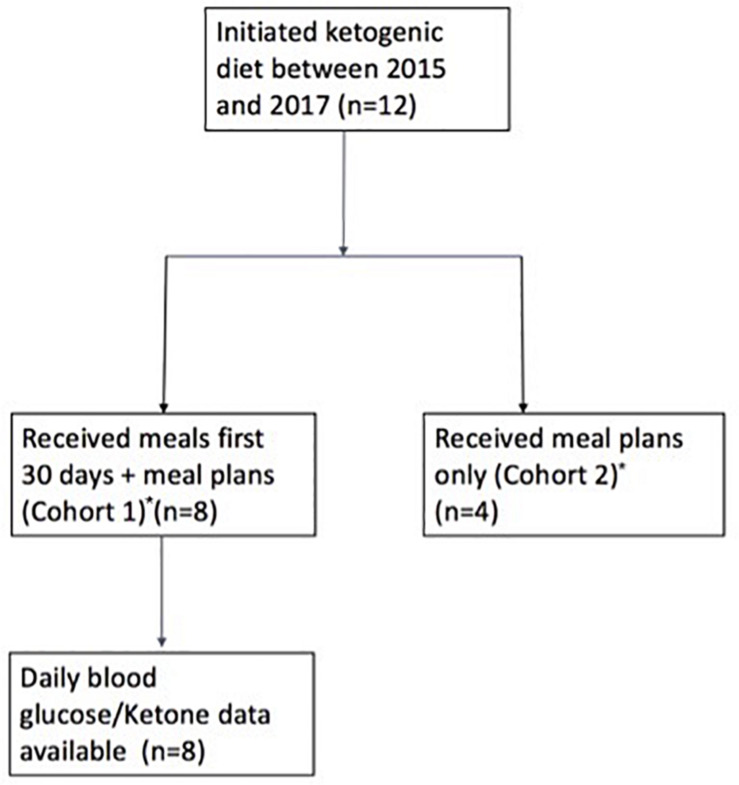
Study flow chart. *Patients belonging to Cohort 1: A–H; Patients belonging to Cohort 2: I–L.

The ketogenic diet was initiated in 12 patients, 8 of whom contributed glucose and ketone data for quantitative analysis (Figure 1). The majority of patients were male (*n* = 10) with a median age of 43.5 (range 27–60) (Table 1). CNS diagnoses included grade 4 GBM (*n* = 6) and grade 2/3 astrocytoma (*n* = 5) and oligodendroglioma (*n* = 1). Patients were receiving standard of care concurrent chemotherapy and radiation therapy (*n* = 10) or supportive care alone (*n* = 2). Patient BMI prior to diet initiation ranged from 21 to 31 kg/m^2^. Two patients reported the use of steroids while on the ketogenic diet. Karnofsky Performance Status (KPS) was fairly well-preserved across all patients at baseline, ranging from 70 to 80. Tumor MGMT methylation status was assessed for seven patients, with methylated MGMT promoter present in four of the seven (Supplementary Table 1).

**TABLE 1:** Patient demographics at time of diet initiation.

ID	Sex	Age	Wt (lbs)	BMI	Race	Diagnosis	Grade	Treatment	Steroid use	KPS
Cohort 1* (Meals provided for first 30 days)										
A	Female	36	197	30	Caucasian	Astrocytoma	2	Supportive Care		70
B	Male	53	165	25	Caucasian	Glioblastoma	4	Bevacizumab + immunotherapy	Yes	80
C	Male	47	176	25	Caucasian	Glioblastoma	4	Dendritic cell vaccination clinical trial + TMZ + RT		80
D	Male	27	229	32	Caucasian	Glioblastoma	4	TMZ	Yes	80
E	Male	45	191	27	Caucasian	Glioblastoma	4	Bevacizumab + TMZ + RT		80
F	Male	36	144	21	Asian	Astrocytoma	2	TMZ + RT		80
G	Male	32	163	22	Caucasian	Oligodendroglioma	2	RT clinical trial + TMZ		90
H	Male	38	154	23	Caucasian	Astrocytoma	3	TMZ (RT		90
**Cohort 2 (Meal plans only)**										
I	Female	42	152	24	Other	Astrocytoma	3	TMZ + RT		
J	Male	59	178	22	Caucasian	Astrocytoma	2	TMZ + RT		80
K	Male	47	172	23	Caucasian	Glioblastoma	4	Clinical Trial (Nivolumab vs. placebo)		80
L	Male	60	174	24	Caucasian	Glioblastoma	4	TMZ + RT		

* Included in quantitative analysis. RT, Radiation Therapy; TMZ, Temozolomide; KPS, Karnofsky Performance Status.

**FIGURE 2:**
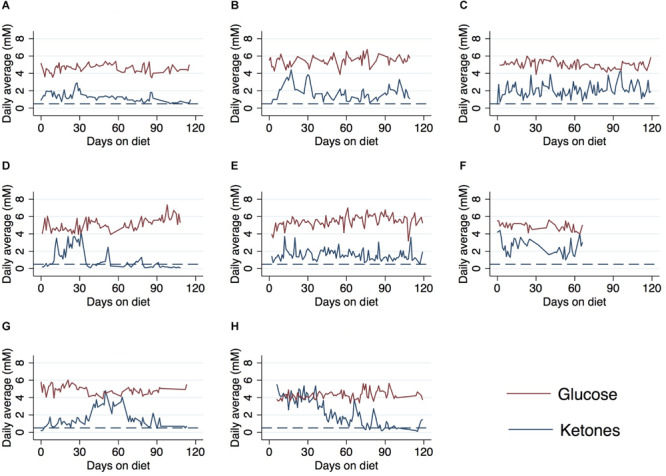
Average daily ketone and glucose levels over the course of a 120-day ketogenic diet in eight patients^∗^. **(A)** Case A, **(B)** case B, **(C)** case C, **(D)** case D, **(E)** case E, **(F)** case F, **(G)** case G, and **(H)** case H. ^∗^Patients from cohort 1 received daily meals for first 30 days on ketogenic diet. Patients F and H already on diet prior to ket/glu measurement and data collection. Reference line (dashed) indicates 0.5 mM threshold for ketosis. Values below line indicate patients not reaching ketosis.

Ketone and glucose levels were reported in eight patients. Baseline ketones ranged from 0.2 to 5.5 mM and fasting and/or random glucose (fasting was not required) ranged from 68 to 99 mg/dL; baseline GKI ranged from 0.67 to 20.5 (Table 2) (some patients were already in ketosis at the time data recording began). Ketone levels increased from baseline in six patients at 30 days, 60 days, and end of study. Blood glucose levels also decreased from baseline in seven of eight patients. The relationship between blood ketone and glucose levels over the 120-study day period for each patient is shown in Figure 2. Peaks in ketones were observed between the 30-day and 60-day period with a general decline toward the end of the study. 30-day GKI ranged from 0.95 to 2.9, while end-of-study GKI ranged from 1.7 to 5.3, possibly reflecting less willingness to strictly follow the ketogenic diet by the end of the 120-day study.

**TABLE 2:** Average daily glucose, Ketone, GKI, and BMI values at baseline, 1 month and end of study in eight patients with evaluable data.

	Baseline				30 Days				End of study^5^			
ID	Glucose (mM)	Ketones (mM)	GKI^1^	BMI^2^ (mg/kg^2^)	Glucose (mM)	Ketones (mM)	GKI^1^	BMI (mg/kg^2^)	Glucose (mM)	Ketones (mM)	GKI^1^	BMI (mg/kg^2^)
A	5.1	0.9	5.7	29.4	4.7	2.3	2.1	28.5	4.4	1.0	4.4	26.9
B^3^	4.8	0.6	8.0	25.3	5.1	3.8	1.3	23.8	5.8	1.1	5.3	23.4
C^4^	4.9	0.7	7.0	24.5	3.9	2.3	1.7	23.4	4.6	2.7	1.7	23.1
D^3^	4.1	0.2	20.5	31.6	4.1	4.3	0.95	31	5.4	0.9	6.0	30.1
E	3.9	1.4	2.8	26.9	5.5	2.3	2.4	25.5	4.7	1.9	2.5	25.5
F^2^	5.5	4.2	1.3	24	4.5	3.2	1.4	22.7	5.0	3.0	1.7	21.0
G	5.8	0.2	28.8	21.6	4.9	1.7	2.9	21.1	5.2	1.7	3.1	18.7
H	3.7	5.5	0.67	21.3	4.1	4.4	0.93	26.9	3.8	1.5	2.6	21.1

*^1^GKI (Glucose Ketone Index), where GKI < 1, therapeutic ketosis; GKI 1-3, high ketosis; GKI 3-6, moderate ketosis; GKI 6-9, low ketosis; GKI > 9 no ketosis. ^2^BMI (body mass index). ^3^Patients B and D were on steroids. ^4^Patients C and F were already on pre-keto diet when starting data collection used for case series. ^5^Day 120, or on last day available data for individual patient if 120 days not reached.*

All patients with available data (*n* = 8) reached ketosis (ketones ≥0.5 mM) during the 120 days from diet initiation (Table 3). Adherence levels of 100% were achieved in five patients and 80–90% in two patients. One patient (Case D) only maintained blood ketone levels >0.5 mM 47% of the study duration and stopped the diet at 79 days. Patients who stopped the diet cited the following reasons: “too restrictive” (*n* = 2), “inconvenient” (*n* = 1), or desire to switch to a modified Atkins diet (*n* = 3). A modified Atkins diet is similar to the ketogenic diet in that it restricts carbohydrate intake, but is more flexible on protein intake and tends to be easier to maintain. Patients who switched to a modified Atkins did so on their own accord for the ease of maintaining during social situations. Diet was continued in five patients beyond the 120-day data collection period. At the time at data cut-off for this retrospective analysis, two patients were deceased due to progressive disease and 10 patients had stable disease (Table 3). Average ketone levels appeared higher in six patients who were alive compared to the two deceased patients (Supplementary Figure 2). Average ketone levels were also slightly higher in unmethylated (*n* = 3) patients compared to methylated (*n* = 4) (Supplementary Figure 3).

**TABLE 3:** Patient outcomes at end of study.

ID	Duration on diet (days)	% of days ketones >0.5 mM^1^	Disease status	Survival status	Reason discontinued
**Cohort 1 (Meals provided)**					
A	>120	100	Stable	Alive	Continuing diet
B	109	100	Progression	Deceased	Diet too restrictive
C	>120	100	Stable	Alive	Continuing diet
D	79	47	Progression	Deceased	Inconvenient with upcoming events
E	>120	100	Stable	Alive	Switched to modified Atkins diet after study
F	67	100	Improvement	Alive	Continuing diet
G	>120	88	Improvement	Alive	Continuing diet
H	120	85	Stable	Alive	Diet too restrictive
**Cohort 2 (Meal plans only)**					
I	>120	N/A	Stable	Alive	Continuing Diet
J	>120	N/A	Stable	Alive	Continuing diet
K	>120	N/A	Stable	Alive	Switched to modified Atkins
L	120	N/A	Stable	Alive	Poor appetite and excess weight loss

*^1^Ad^1^adherence is defined by percentage of days where ketone levels >0.5 mmol/dL of total number of study days with available ketone and glucose data. N/A: Missing ketone and glucose measurements for patient. Patient F continuing diet beyond data collection period.*

**FIGURE 3:**
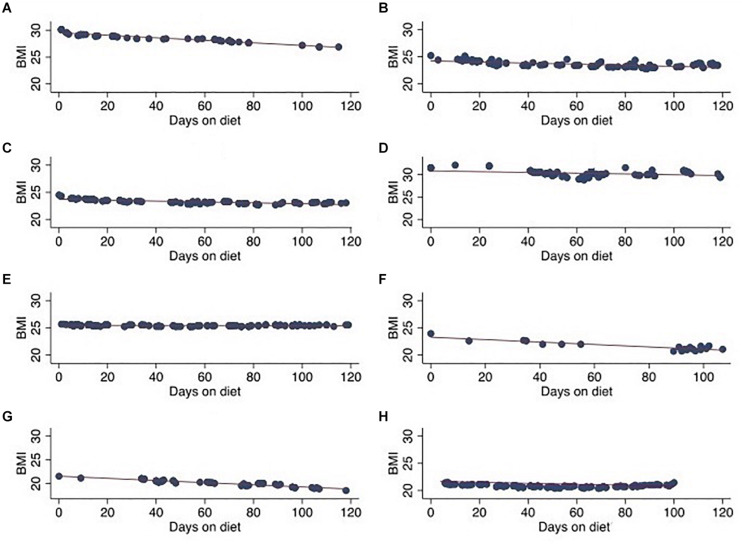
Patient BMI over 120-day ketogenic diet period in eight patients. **(A)** Case A, **(B)** case B, **(C)** case C, **(D)** case D, **(E)** case E, **(F)** case F, **(G)** case G, and **(H)** case H.

Feasibility and safety were evaluated by monitoring patient weight and BMI throughout the duration of the ketogenic diet. All patients experienced some degree of weight loss and decreased BMI when on the ketogenic diet. A >10% difference in BMI was observed in three patients at end of the study period. Most patients (*n* = 5) lost between 5% and 10% of their initial BMI at end of study period with three patients experiencing >10% difference in BMI and one patient with stable weight and BMI (Table 2 and Figure 3).

Overall, patient symptoms improved including increased energy levels, physical mobility, mood, and cognitive function as reported in the clinician notes and ESAS assessments, where available (Table 4). Notable symptoms included fatigue, which improved in five patients, increased in three patients, was stable in three patients and absent in one. Increasing severity of headaches was reported in two patients, was unchanged from baseline in five patients, and improved in one. Appetite decreased in five patients and improved in four (not reported in three). Seizures improved in two patients and increased in severity in one patient. No other patients experienced any seizures throughout the duration of the diet.

**TABLE 4:** Reported symptoms in 12 patients from initiation of diet to end of 120-day study period.

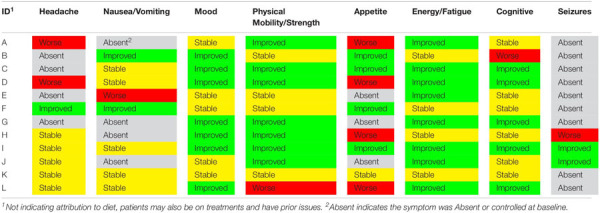

### Case Presentations

#### Cohort 1 (Meal Plans Provided for First 30 Days):

##### Case A

A 38-year-old female was diagnosed with a grade 2 spinal cord diffuse astrocytoma following partial resection in September 2012. She initially presented in 2008 with left lower extremity parasthesias and was found to have a non-enhancing intramedullary spinal cord lesion at the T11 level at that time. Post-operative treatment with radiation therapy was quickly aborted due to worsening right lower extremity weakness. Because the patient was unable to tolerate radiation therapy, chemotherapeutic treatment with temozolomide and subsequently bevacizumab was initiated. Both of these treatments were also discontinued due to poor tolerability and lack of symptomatic or radiographic benefit. At the initiation of the ketogenic diet pilot project, the patient was overweight (BMI: 30.4) and had intractable coccyx pain. Patient followed a 3:1 standard ketogenic diet. Patient’s weight decreased within the first 3 months. Patient reported that changes to meal plan helped immensely with positive outlook and appetite/digestion. Follow-up imaging showed a reduction in the size of the predominantly non-enhancing mass, as well as resolution of peripheral enhancement seen on the pre-diet scan. Patient continues to do well on a ketogenic diet.

##### Case B

A 52-year-old male was diagnosed with a left temporal GBM in November 2013 after presenting with memory deficits and aphasia. He was initially treated with standard radiation and temozolomide. At the time of ketogenic diet therapy, the patient was on fifth-line treatment for recurrent GBM, consisting of the antiangiogenic therapy bevacizumab (which is FDA-approved for recurrent GBM) in combination with irinotecan. Patient had nausea, headaches, and fatigue prior to starting diet. Patient followed ketogenic diet for 3.5 months. Patient started to get restless on diet and craved carbs and sweets; however, the patient did report being able to add physical exercise to his routine along with dance lessons. After discontinuation of ketogenic diet, patient reported worsening symptoms (including difficulty reading and decreased short term memory). Deceased January 2017.

##### Case C

A 47-year-old male was diagnosed with left parietal GBM in July 2015 after presenting with expressive aphasia. He enrolled in a clinical trial consisting of dendritic cell vaccine treatment in addition to standard chemoradiation. Patient’s pre-diagnosis diet was “paleo.” Patient began a 3:1 ketogenic diet. After 2 months on a ketogenic diet, short-term memory improved and the patient regained the ability to read. MRI demonstrated diminished contrast enhancement. After 6 months, patient reported feeling great and with no interest in stopping the diet. Patient continues on 3:1 ketogenic diet.

##### Case D

A 27-year-old male was diagnosed with a left parietal GBM in February 2013 after presenting with seizure. At the time of ketogenic diet initiation, he was being treated for third recurrence with a bevacizumab-based regimen. The patient had a longer transition into ketosis, possibly related to previously being on a vegan diet and concomitant treatment with dexamethasone. One month following diet initiation, the patient reported improved energy, aphasia, and reading ability. He started a strength-training program and began participating in gaming tournaments again. MRI at this time demonstrated diminished contrast enhancement. Patient stopped diet after 79 days due to challenges with adherence during social events. Patient died 1 year after starting diet (September 2016).

##### Case E

A 45-year-old male was diagnosed with a right frontal grade 2 diffuse astrocytoma in March 2005. He was followed without treatment until presenting with headaches in December 2011, at which time biopsy demonstrated transformation to GBM (grade 4). At the time of ketogenic diet initiation in May 2016, he was being treated with bevacizumab for recurrent disease. Within the first 2 months, the patient reported improvements in short-term memory and began a strength-training program. Patient continued with the ketogenic diet for 120-days before transitioning to a modified Atkins diet which he continues on at this time.

##### Case F

A 36-year-old male was diagnosed with a left temporal grade 2 astrocytoma grade in August 2017 after presenting with a seizure. He was treated with radiation and temozolomide, and began the ketogenic diet in September 2017. Prior to diagnosis, the patient was vegetarian. Patient started on 2.5:1 ketogenic diet, and subsequently reported improvement with headaches, nausea, and appetite. He was able to return to work and began jogging again. He continues on the ketogenic diet, although only recorded approximately 2 months of quantitative ketone and glucose data.

##### Case G

A 32-year-old male was diagnosed with a right frontal grade 2 oligodendroglioma in March 2016 after presenting with seizure. Alongside ketogenic diet initiation, he was treated with radiation and temozolomide chemotherapy. Brain MRI performed July 2016 demonstrated a partial response of non-enhancing FLAIR hyperintense disease. For the first 3 months of treatment, the patient reported symptoms of severe constipation, dizziness, and fatigue. Weight was closely monitored as the patient’s initial BMI was 22, By August 2016, patient felt much more comfortable on the ketogenic diet and reported improved mental clarity. He was able to resume work as well as his recreational hobbies. Patient continues on the ketogenic diet.

##### Case H

A 38-year-old male was diagnosed with a left frontotemporal non-enhancing grade 3 anaplastic astrocytoma by August 2014 after presenting with seizure. He received standard post-operative treatment with concurrent radiation and temozolomide, followed by 12 cycles of adjuvant temozolomide. At the time of ketogenic diet initiation, the patient was being monitored off therapy, but was still experiencing significant fatigue and forgetfulness that limited his ability to work. He reported improved mood and strength while on the diet, but discontinued after 4 months because he felt the diet was too restrictive.

#### Cohort 2 (Meal Plans Only)

##### Case I

A 41-year-old female was diagnosed with a right temporal grade 3 anaplastic astrocytoma in February 2017 after presenting with seizures that were initially misdiagnosed as panic attacks. Patient started a 3:1 ketogenic diet approximately 1 month after diagnosis while being treated with radiation and temozolomide. Prior to diet initiation, the patient experienced frequent breakthrough seizures and significant treatment-related fatigue. Seizures stopped and fatigue improved significantly shortly after diet initiation. She was able to resume playing recreational sports again and continues on a ketogenic diet.

##### Case J

A 58-year-old male was diagnosed with a left temporal grade 2 diffuse fibrillary astrocytoma in July 2016 after presenting with seizure. Patient started on a strict 3:1 ketogenic diet in April 2017. By the end of April, breakthrough seizures, which had been numerous, ceased. Valproic acid was decreased with no further seizures. Patient also reported improvement in fatigue, and decreased hand tremor. Patient continues on a ketogenic diet.

##### Case K

A 47-year-old male was diagnosed with a right occipital GBM after noting visual disturbances in April 2017. He enrolled in a clinical trial of plus nivolumab immunotherapy vs. placebo, with both arms receiving standard chemoradiation. Ketogenic diet was initiated in May 2017, ranging from 2:1 to 3:1. Two months after ketogenic diet initiation, patient reported that improved fatigue and was able to taper his methylphenidate dose. In October 2017 (>120 days after diet initiation), he decided to follow a modified and less restrictive Atkins diet.

##### Case L

A 60-year-old male was diagnosed with a right temporal GBM in July 2016 after presenting with seizures. He was treated initially with standard radiation and temozolomide, and adopted a ketogenic diet in September 2016. By the end of the month, shortly after ketogenic diet initiation, he reported symptoms generally associated with treatment (radiation and chemotherapy), including fatigue, weakness, and loss of appetite. Two months later, the patient reported feeling much better. Weight stabilized, and he started walking 45 min daily. He began adjuvant cycles of temozolomide chemotherapy, which he tolerated well. Patient continues on a ketogenic diet.

## Discussion

In this retrospective case series analysis, we describe the experience of 12 patients with CNS malignancies who incorporated the ketogenic diet into their treatment plan. Eight patients participated in a pilot project that included the option of receiving proprietary prepared meals, and 4 patients adopted the ketogenic diet without prepared meals and with meal plans alone. All patients worked closely with oncology dietitian to develop individually tailored meal plans. Blood glucose and ketone levels were monitored twice daily, as were body weight and body composition, clinical symptoms, and potential adverse effects. Clinical outcomes that were tracked include qualitative assessments of quality of life and neurocognitive function, seizure control, radiographic response, time-to-progression, and survival (Supplementary Figure 1).

All patients were adherent and were able to maintain average daily blood ketone levels greater than or equal to 0.5 mM, which is typically recognized to be the threshold for nutritional ketosis. The ketogenic diet was generally well tolerated, with eight patients continuing with the diet or a low-carb, modified version of the diet on their own beyond the 120-day study period. As expected, glucose and ketone levels were observed to be inversely related, and patients who initiated a ketogenic diet or low-carb diet prior to the first blood ketone and glucose measures (Patients F and H) were able to reach ketosis very quickly (within the first week).

**FIGURE 4:**
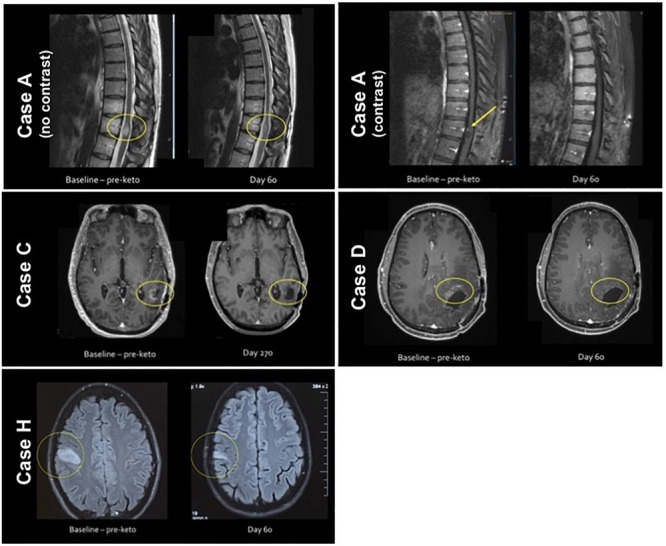
Visual disease assessment pre and during ketogenic diet.

Notable differences in energy, mood, neuro-cognitive function and overall well being were observed in a qualitative assessment of the patients’ symptoms. In some cases, this may be due to a direct effect of the ketogenic diet on physiology; in others, it may reflect improved tolerability of chemotherapy and radiation therapy due to the diet. Findings also suggest the ketogenic diet may assist with seizure control; the majority of patients who previously experienced seizures experienced a decrease in the number of seizures for the duration of the diet, and some patients were able to decrease the dosage of seizure medication. In four patients, imaging demonstrated reduction in contrast enhancement or T2 hyperintensity suggestive of radiographic response; in three of these patients, the ketogenic diet was instituted concurrently with chemotherapy (although for this patient population, radiographic responses to chemotherapy are relatively infrequent), and in one patient, radiographic response was noted following institution of the ketogenic diet in the absence of other treatment (Figure 4). Primary safety concerns of the ketogenic diet in this population pertained to weight loss and appetite, and meal plans and caloric goals were adjusted to maintain healthy weight and body composition.

Despite the explosion of interest in the ketogenic diet in the brain tumor patient community, to date, the medical literature regarding use of a ketogenic diet for patients with CNS malignancies has been limited. Nebeling et al. (1995) reported positive responses in two children with astrocytoma. Zuccoli et al. (2010) reports on a single case report of a patient diagnosed with GBM receiving standard therapy and following a restricted ketogenic diet. Schmidt et al. (2011) conducted a pilot study on 16 patients with advanced cancer which concluded that the ketogenic diet was tolerable with no severe adverse effects. More recently, a small clinical trial of 11 patients with newly diagnosed GBM was performed in the Netherlands. Of the 11 patients who started the study protocol, nine reached ketosis and six completed the study period of 14 weeks. Overall survival ranged from 9.8 to 19.0 months. The authors noted that a supportive partner and intensive counseling were essential for coping (Perez et al., 2019; van der Louw et al., 2019). In a recent single-center trial performed in the United Kingdom, 12 patients with newly diagnosed GBM were randomized 1:1 to receive either a modified ketogenic diet or medium chain triglyceride ketogenic diet. Recruitment rate was low, and only four patients completed the 3-month diet (Martin-McGill et al., 2020). Studies are further summarized in recent systematic reviews on this topic (Martin-McGill et al., 2018b; Schwartz et al., 2018).

Although the data described in this report are limited by the retrospective case series design, it is worth noting that the quantity of data and close monitoring of patients performed in this study is quite robust compared to most of the studies in the published literature regarding the ketogenic diet. Indeed, the close tracking of point-of-care and patient-entered data used to monitor this cohort of patients has been largely feasible due to recent technological advancements, such as online data management tools and home blood ketone monitoring (which provide more accurate readings than urine ketone strips). Close tracking of blood glucose and ketone levels also provides strong internal validation of dietary adherence, and mitigates concerns related to the validity of patient reporting that can affect diet studies.

Several limitations of this analysis relate to the case series design. Given the retrospective nature of the report, some data were missing from selected patients, particularly in Cohort 2. Additionally, patients included in this case study were motivated, interested in adopting a ketogenic diet, and generally had good functional status at baseline. These factors may differ in an unselected patient population. Close monitoring and data collection may also have impacted patient adherence. Conclusions regarding potential therapeutic effects are limited by small sample size, concurrent treatment, and lack of uniformity regarding diagnosis and stage of disease. On the other hand, the fact that patients with a wide array of diagnoses were all able to tolerate a ketogenic diet speaks to the general feasibility of this approach. Finally, although the commonly accepted metric of 0.5 mM was used as a threshold for determining nutritional ketosis, the ideal therapeutic range for ketosis in this patient population – if it exists – has yet to be elucidated.

Despite these limitations, the results of this case series and others should be further explored in the setting of prospective clinical trials. Findings from our study may inform the design of future studies; in particular, our experience has shed light on the importance of tracking neurocognitive function and quality of life for patients on a ketogenic diet, as well as the feasibility of tailoring meal plans based on daily assessments of blood glucose and ketone levels, body weight and composition, and clinical symptoms. Compared to two recent small trials that incorporated a ketogenic diet for patients with GBM, adherence and ability to achieve and maintain ketosis in this patient cohort was comparatively straightforward (Martin-McGill et al., 2018a; van der Louw et al., 2019). One possible explanation is that patients that chose to participate in this study were generally clinically stable, and had already had some time to come to terms with their diagnosis. For patients in Cohort 1, it is also possible that having pre-packaged meals simplified the initial process of achieving ketosis, even though most patients eventually transitioned to home-prepared meals.

We are currently exploring the feasibility and safety of the ketogenic diet in a phase 1 clinical trial (NCT03451799) for patients with recently diagnosed GBM. In addition to assessing feasibility, safety, and clinical outcomes, this trial will provide a wealth of additional data in the form of in-depth metabolic profiling, microbiome analysis, and tumor analysis. There are also other ongoing studies currently evaluating the ketogenic diet (NCT03328858, NCT03160599, NCT02694094) at other institutions. Clinicaltrials.gov currently lists over 100 trials looking at the ketogenic diet and approximately 12 of those trials include conditions related to CNS malignancies.

There is also great interest in extending metabolic therapy beyond utilization of a ketogenic diet alone. One option is to supplement the diet with exogenous ketones, either in salt or ester form, with the idea that higher levels of ketosis may have a greater antineoplastic effect. At this time, it is still unknown whether elevating ketone levels with exogeneous supplementation provides the same level of benefit as diet-induced ketosis. Another area of interest is combining the ketogenic diet with medications such as metformin or compounds that restrict glutamine availability in order to further increase metabolic stress on tumor cells (Seyfried et al., 2017). The optimal combination of therapies is not known at this time.

This case series contributes to the growing body of literature suggesting that a ketogenic diet is safe and feasible in patients with CNS malignancies. Radiographic responses were noted in several patients, although in most instances the diet was being administered along with concomitant therapy. In addition to the ongoing phase 1 trial at our institution, several other trials are also ongoing. We anticipate proceeding with a phase 2 trial powered to detect efficacy in the near future.

## Conclusion

In conclusion, the ketogenic diet was feasible in this cohort of patients with CNS malignancies. The benefits that were noted in this patient cohort were intriguing and not easily explained by mechanisms other than effects from the ketogenic diet. These effects warrant further examination in the form of prospective clinical trials.

## Data Availability Statement

The raw data used and/or analyzed supporting the conclusions of this article will be made available by the authors, without undue reservation, to any qualified researcher.

## Ethics Statement

The studies involving human participants were reviewed and approved by Cedars Sinai Medical Center, Office of Research and Compliance (institutional reference number: Pro00049543). Written informed consent for participation was not required for this study in accordance with the national legislation and the institutional requirements.

## Author Contributions

CP collected and analyzed the data, and was a major contributor in writing the manuscript. GG analyzed and interpreted the patient data, as well as contributed to writing the manuscript. LA also interpreted the patient data, assisted in data collection, and wrote the case summaries. JH designed the experiment and modified the manuscript. All authors read and approved the final manuscript.

## Conflict of Interest

The authors declare that the research was conducted in the absence of any commercial or financial relationships that could be construed as a potential conflict of interest.

## Abbreviations

BMI: body mass index
ESAS: Edmonton Symptom Assessment Scale
GBM: glioblastoma
GKI: glucose ketone index.
