# Corrigendum: Exploring the Feasibility and Effects of a Ketogenic Diet in Patients With CNS Malignancies: A Retrospective Case Series

**DOI:** 10.3389/fnins.2020.00661

**Published:** 2020-06-25

**Authors:** Cristina M. Panhans, Gillian Gresham, L. J. Amaral, Jethro Hu

**Affiliations:** Cedars-Sinai Medical Center, Samuel Oschin Comprehensive Cancer Center, Los Angeles, CA, United States

**Keywords:** ketogenic diet, glioma, glioblastoma, astrocytoma, Warburg effect, Atkins, ketosis, metabolic therapy

An author's name was incorrectly spelled as “J. L. Amaral.” The correct spelling is “L. J. Amaral.”

In addition, there was a mistake in the legend for Figure 2. The reference line that indicates 0.5 mM threshold is “(dashed)” and not “(red).” The correct legend appears below.

**FIGURE 2 |** Average daily ketone and glucose levels over the course of a 120-day ketogenic diet in eight patients^*^. **(A)** Case A, **(B)** case B, **(C)** case C, **(D)** case D, **(E)** case E, **(F)** case F, **(G)** case G, and **(H)** case H. ^*^Patients from cohort 1 received daily meals for first 30 days on ketogenic diet. Patients F and H already on diet prior to ket/glu measurement and data collection. Reference line (dashed) indicates 0.5 mM threshold for ketosis. Values below line indicate patients not reaching ketosis.

The authors apologize for this error and state that this does not change the scientific conclusions of the article in any way. The original article has been updated.

