# Supplementary material for: Exploring the Feasibility and Effects of a Ketogenic Diet in Patients With CNS Malignancies: A Retrospective Case Series
Source: Front Neurosci. 2020 May 19;14:390. doi: 10.3389/fnins.2020.00390 (PMC7248360; doi:10.3389/fnins.2020.00390)
Supplement: Supplementary file 1 [file Data_Sheet_1.docx]

Supplementary Material

**Supplemental Table 1.** Laboratory and Molecular Findings

| **ID** | **Diagnosis** | **Methylation Status** | **IDH1 R132H** | **EGFR** | **PTEN** | **1p/19q deletion** |
| --- | --- | --- | --- | --- | --- | --- |
| A | Astrocytoma | Unmethylated | Negative | Not amplified | Retained | - |
| B | Glioblastoma | Methylated | Negative | Amplified | Retained | - |
| C | Glioblastoma | Methylated | Negative | Amplified | - | Retained |
| D | Glioblastoma | Methylated | Positive | Not amplified | Loss | Retained |
| E | Astrocytoma | Methylated | Positive | - | Loss | - |
| F | Oligodendroglioma | Unmethylated | Positive | Amplified | - | Retained |
| G | Glioblastoma | - | Positive | - | - | Co-deletion |
| H | Glioblastoma | Unmethylated | Positive | - | - | Retained |
| I | Glioblastoma | Methylated | Positive | Not amplified | - | - |
| J | Astrocytoma | Indeterminate | Negative | Amplified | Loss | Loss |
| K | Astrocytoma | Methylated | Negative | Amplified | - | - |
| L | Astrocytoma | Unmethylated | Negative | Amplified | Loss | - |

IDH1 (Isocitrate dehydrogenase 1)

EGFR (epidermal growth factor receptor)

PTEN (Phosphatase and tensin homolog)

1p/19q (1p/19q co-deletion between chromosomes 1 and 19)

**Supplemental Figure 1.** GKI (Glucose-ketone index) Over 120-day Ketogenic Diet in 8 Patients

**
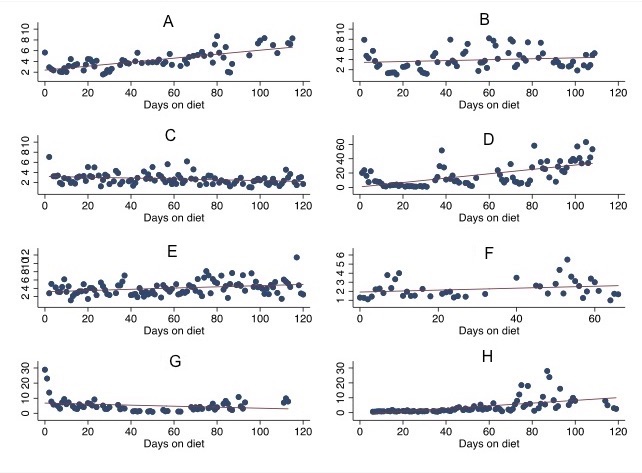
**

**Supplemental Figure 2.** Ketone Levels by Survival Status in 8 Patients

**Supplemental Figure 3.** Ketone Levels by Methylation Status in 7 Patients
